# Immunoprevention of KRAS-driven lung adenocarcinoma by a multipeptide vaccine

**DOI:** 10.18632/oncotarget.19831

**Published:** 2017-08-01

**Authors:** Jing Pan, Qi Zhang, Shizuko Sei, Robert H. Shoemaker, Ronald A. Lubet, Yian Wang, Ming You

**Affiliations:** ^1^ Cancer Center and Department of Pharmacology & Toxicology, Medical College of Wisconsin, Milwaukee, WI, USA; ^2^ Chemopreventive Agent Development Research Group, Division of Cancer Prevention, National Cancer Institute, Bethesda, MD, USA

**Keywords:** immunoprevention, KRAS, lung adenocarcinoma, peptide vaccine, MHC class II

## Abstract

Lung cancer remains the leading cause of cancer death worldwide. Mutations in KRAS are detected in up to 30% of lung cancer cases. No effective therapies specifically targeting mutant KRAS have been developed. Vaccination against KRAS mutants is one of the venues of active exploration. The present study evaluated both immunogenicity and antitumor efficacy of a newly formulated multipeptide vaccine targeting multiple epitopes of the KRAS molecule. The formulated vaccine contained top four peptides, which elicited the strongest immunologic response and showed 100% sequence homology between human and mouse. The multipeptide KRAS vaccine was tested in an inducible CCSP-TetO-KRAS^G12D^ mouse model, where the vaccine was administered prior to activating the mutant KRAS protein. The KRAS peptide vaccine exhibited striking efficacy, reducing tumor number and tumor burden by >80% when compared with adjuvant alone. Splenocytes collected from vaccinated animals showed a robust immunologic response to the immunizing peptides. Furthermore, *in vitro* stimulation of these splenocytes by the vaccinated peptides resulted in the secretion of cytokines indicative of Th1 responses but with minimal secretion of Th2-related cytokines. The multipeptide KRAS vaccine was immunogenic and efficacious in the primary prevention of KRAS-induced lung cancer, indicating that the approach potentially can be used to prevent other KRAS-driven cancers, either alone or in combination with other modalities.

## INTRODUCTION

Lung cancer, which has a low five-year survival rate, remains the leading cause of cancer death worldwide, with cases rising globally [1]. New approaches are needed to improve the clinical outcome for these patients. A number of genetic and epigenetic abnormalities are identified as essential drivers promoting tumor development [2, 3]. This has facilitated the identification and characterization of potential tumor antigens that have become relevant targets for the development of cancer vaccines. Among the oncogenes in NSCLC (non-small cell lung cancer), mutations of the Kirsten rat sarcoma viral oncogene homolog (KRAS) are most frequently observed. They may represent up to 30% of lung cancer in the Caucasian smoking population [4], with 80% of them are specifically altered in codon12 [5]. However, unlike EGFR mutation, there’s no specific targeted therapies have been developed for KRAS mutations yet, partially due to the lack of druggable pockets and cavities on the RAS surface [6], except for the new compounds recently discovered that specifically target mutant KRAS^G12C^ [7], the development of alternative therapies or preventive measures has great appeal.

One potential approach to prevent cancer development is through the use of vaccines [8–10]. Vaccination against KRAS mutants is one of the venues of active exploration [11]. GI-4000 are intact, heat-inactivated yeast containing known KRAS mutations, and have been shown to be well tolerated and able to stimulate mutation-specific T-cell responses, the cytotoxic response in multiple clinical and preclinical tests, which ultimately lead to favorable overall survival trend [12–14]. However, whole organism vaccines are still limited by the difficulties in large-scale manufacturing, as well as repeat dosing to enhance vaccine efficiency [15]. Compare to conventional vaccines, advantages of immunoprevention using peptide vaccines include minimal toxicity, immediate and long-term memory, ease of delivery and cost effectiveness [16]. Peptide vaccination against tumor-associated antigen as a means of treating cancer patients or preventing the development of tumors in high-risk individuals (e.g., former or current smokers) is currently an area of intense research [17]. Peptide vaccines are designed to prime the immune system to attack tumor cells which overexpress both normal and mutant proteins. They can elicit memory T cells that remain in lymph nodes until exposed to the target antigen. After stimulation, T cells migrate to the site of antigen-expressing lesions regardless of the location and will proliferate and destroy those lesions. Numerous investigators have attempted to inhibit mutant KRAS, often employing relatively short MHC class I restricted peptides with minimal to moderate success [18, 19]. We, however, have taken a rather different approach by employing longer peptides with predicted binding affinity to MHC class II. Using this approach, we and our collaborators have previously developed a multipeptide multivalent vaccine that elicited robust Th1 immune responses and effectively blocked the development of neu-driven mammary tumors [9], or mutant EGFR1-driven lung cancer [10]. These studies show that one can prime the development of robust immune response against a particular epitope/peptide, or generate effective MHC class II-mediated immune responses against a variety of target peptides despite potential tolerance.

In the present study, we evaluated both immunogenicity and antitumor efficacy of this newly formulated multipeptide (peptides 15–17 amino acids long) vaccine targeting multiple epitopes of the KRAS molecule in a mouse model of a KRAS-driven lung tumor [20]. We demonstrate that the multipeptide KRAS vaccine is immunogenic and efficacious in the primary prevention of KRAS-induced lung cancer, indicating that the approach potentially can be used to prevent other KRAS-driven cancers, either alone or in combination with other modalities.

## RESULTS

### Identifying KRAS peptides that elicit T cell response by a multi-scoring system combining multiple MHC class II peptide binding algorithms

**Figure 1:**
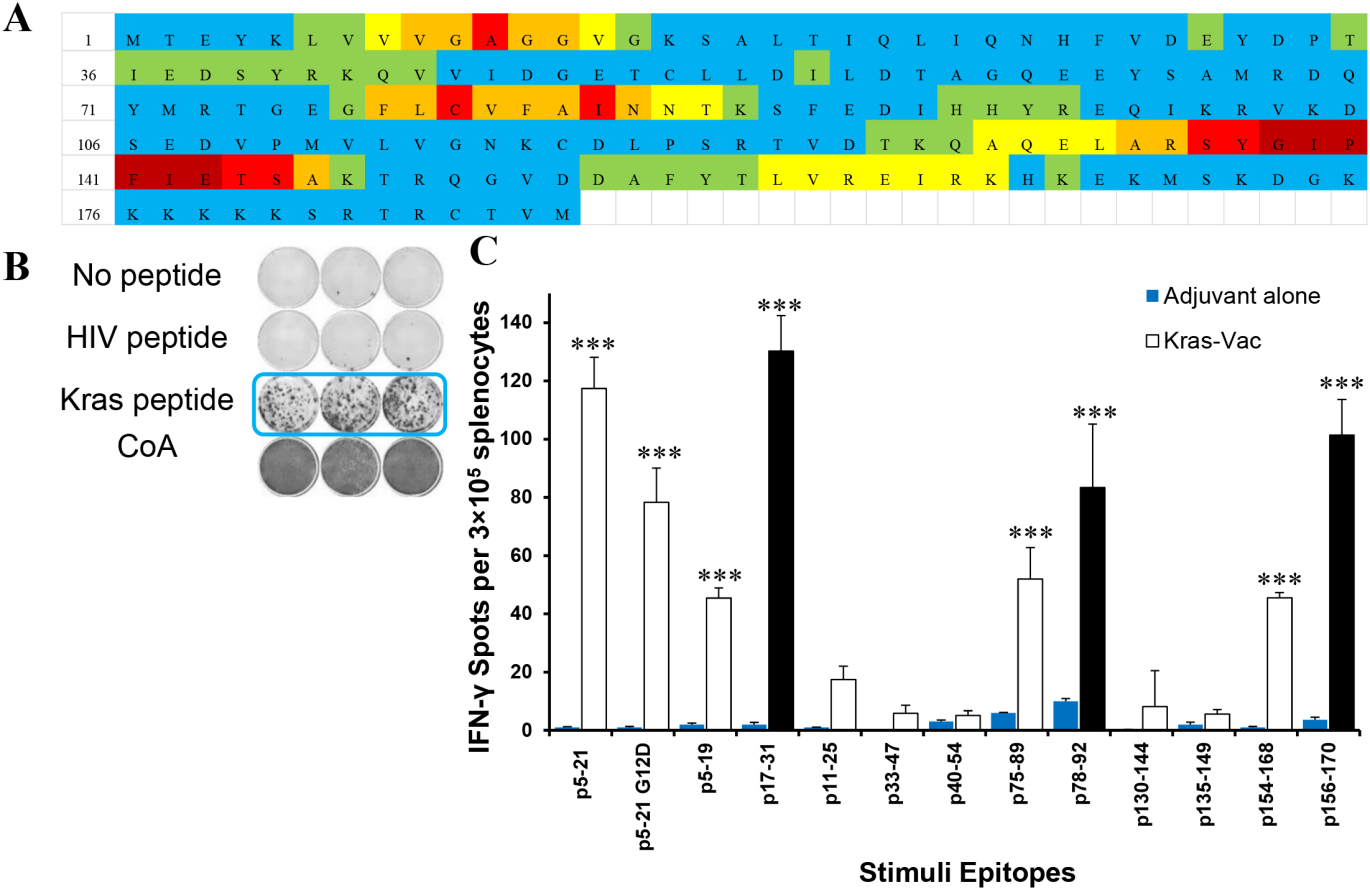
*In vivo* screen for peptide candidates by ELISPOT in naïve mice **(A)** Immunogenic heatmap for identifying peptides associated with highest binding affinity across multiple MHC class II alleles. Colors represent percentile to highest score from three algorithms for each amino acid from dark red to light blue in the order of rank scores. Color strata are as follows: dark red ≥ 75% of the highest score; red = 50∼75% of the highest score; orange = 40∼50% of highest score; yellow = 30∼40% of the highest score; green = 20∼30% of the highest score; blue ≤ 20% of the highest score. **(B)** Representative ELISPOT results showing T cell responses to specific KRAS peptides from mouse splenocytes stimulated with no antigen (first row), negative control peptide (HIV peptide, second row), target peptide (third row), or positive control ConA (fourth row). **(C)** Quantified ELISPOT results. Mice were vaccinated with each single KRAS peptide (sequence listed in Supplementary Table 1). Then, splenocytes were collected and pulsed with no antigen control, each single KRAS peptide, or negative control peptide (HIV peptide), or positive control ConA. After 72h of incubation, the ELISPOT assay was performed, plates were scanned, and spot numbers were statistically analyzed. Open bar, ELISPOT reads from KRAS peptide vaccinated animals pulsed with specific KRAS peptide; blue bar, ELISPOT reads from animals injected only with adjuvant and pulsed with specific KRAS peptide; black bar, ELISPOT reads from three most significant KRAS peptide vaccinated animals. Data are shown as the mean ± SE of three replicate wells per group, n=5, ***P<0.001.

Using a multi-scoring system that combines multiple MHC class II peptide binding algorithms, immunogenic “hotspots” were identified; 11peptides (Supplementary Table 1) were selected; and their immunogenicity was evaluated in naïve mice using the IFN-γ ELISPOT assay. The KRAS 17-mer peptide (p5–21) that covers the mutation in codon 12, and its G12D mutant (p5–21 G12D) were also selected as robust peptides to stimulate immune response [22]. Figure 1A demonstrates the entire KRAS protein sequence and the immunogenic “hotspots” identified through the multi-scoring system solely on MHC class II epitopes. Of the 11 newly designed peptides, six of them (45%) were immunogenic, with p5–21, p17–31, p78–92, and p156–170 eliciting the strongest IFN-γ response (Figure 1C). Interestingly, p75–89, which differed from p78–92 by only three amino acids, yielded a much weaker response in naïve animals. Similarly, p154–168 yielded a much weaker response than p156–170 despite great overlap in the peptides, and p5–19, which is contained within p5–21 with only two amino acids missing in the C-terminus, yielded less than 50% response of p5–21. The two 17-mer peptides (p5–21 and p5–21 G12D mutant) also demonstrated strong immunogenicity with a mean for the IFN-γ-secreting cells of around 100 spots per well (SPW) compared to10 SPW for the negative control HIV peptides (Figures 1B, 1C). As expected, mice immunized with adjuvant alone did not develop any antigen-specific IFN-γ response to either single peptide or multipeptide stimulation, with the mean IFN-γ response similar to that of the HIV peptide (*P*<0.001, Figure 1C). To include peptides against both wild-type and mutant KRAS, also based on a high degree of homology between human and mouse KRAS, peptides p5–21, p5–21 G12D, p17–31, and p78–92 were chosen to formulate a multipeptide KRAS vaccine in the preventive efficacy study.

### KRAS-specific multipeptide vaccine prevents lung tumor formation in primary prevention setting

**Figure 2:**
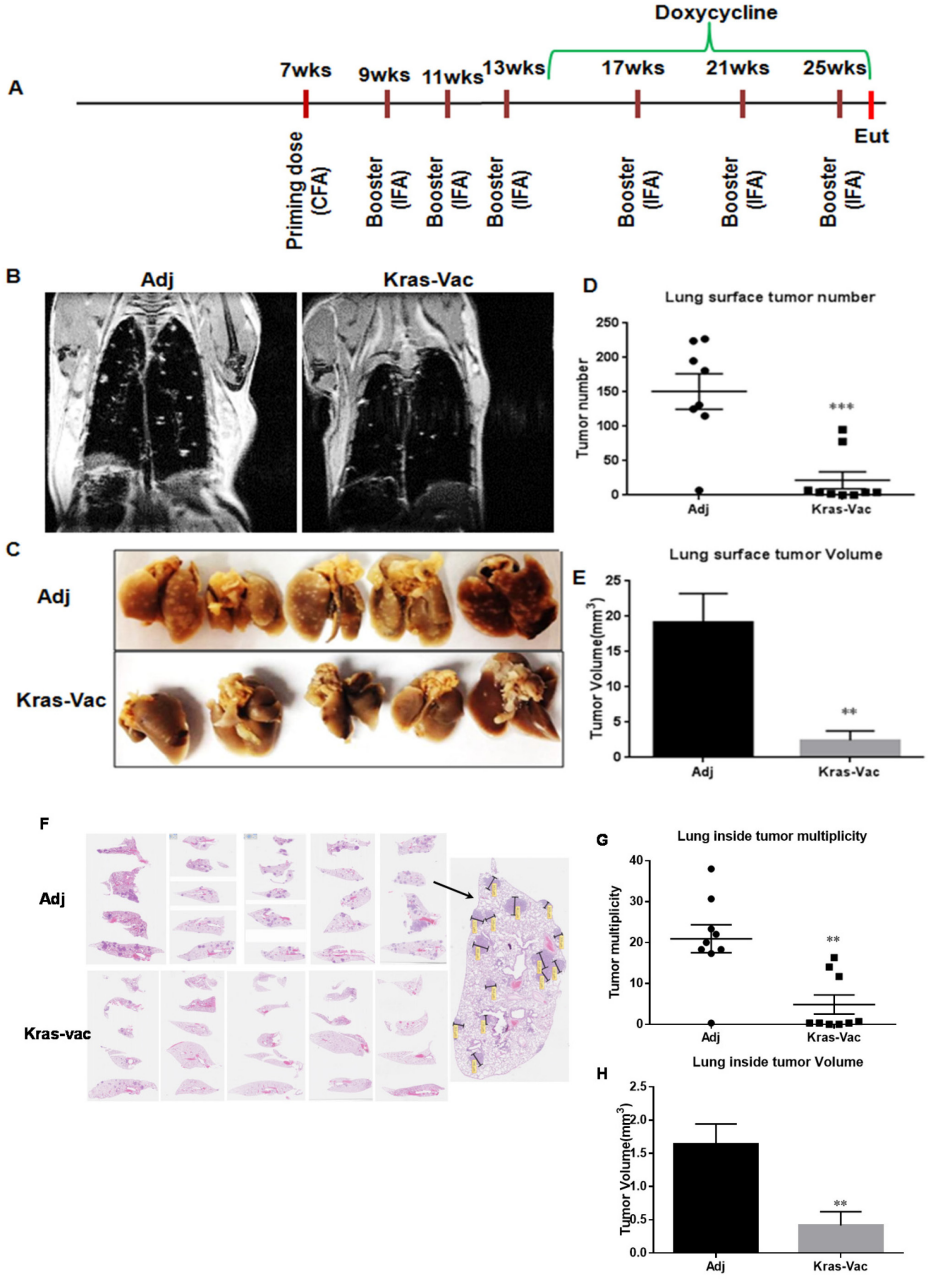
KRAS vaccine inhibited KRAS^G12D^-driven mice in conditional CCSP-KRAS mice in prevention setting **(A)** Schematic of the experimental design outlining timing of vaccine administration, induction of the oncogenic transgene, and experimental endpoint. **(B)** Representative MRI images from mice injected with either adjuvant control or KRAS multipeptide vaccine. Quantification of surface tumor. Typical lung lobes with surface tumors from each group **(C)**, surface tumor number **(D)**, and tumor volume quantitation **(E)**. **(F)** Representative lung lobes (H&E slides) to show tumors inside lungs from each group, illustration of tumor counting. Inside tumor number **(G)** and tumor volume quantitation **(H)**. Data are shown as the mean ± SE, n=8 (adj), n=9 (KRAS-Vac), **P<0.01, ***P<0.001. CFA, Complete Freund’s Adjuvant; IFA, Incomplete Freund’s adjuvant; Eut, euthanization.

We conducted the experiment in a doxycycline-inducible KRAS^G12D^ murine model. As illustrated in Figure 2A, the first prime vaccination was given with CFA as adjuvant when mice were seven weeks old, followed by three boosting vaccinations administered with IFA at two-week intervals. One week after the last vaccination in IFA, doxycycline was started in diet to induce KRAS^G12D^ expression. Additional boosting vaccinations were administered at four-week intervals throughout the rest of the study. Tumor growth was first evaluated *in situ* with MRI imaging from at least three representative animals just prior to the experimental endpoint. As shown in Figure 2B, MRI imaging demonstrated significant qualitative differences within the lung parenchyma between non-vaccinated and vaccinated mice. Representative non-vaccinated and vaccinated mouse lungs are shown in Figure 2C. Lungs from non-vaccinated mice were fully covered with lung adenocarcinoma, whereas lungs from KRAS-specific peptide vaccinated mice appeared virtually free of gross tumors. KRAS vaccine significantly decreased surface tumors from ∼150 tumors per mouse lung in non-vaccinated mice to ∼21 tumors in vaccinated mice (Figure 2D), and decreased tumor volume nearly 90% (Figure 2E, 19.1mm^3^ to 2.4mm^3^). Further analysis was also done on the internal tumor counting via H&E staining. A representative histological examination of lungs from vaccinated versus adjuvant-treated animals reveals changes within the lung parenchyma (Figure 2F). Similar to surface tumor counting, vaccinated animals demonstrated an average of ∼5 tumors per slide with average volume of 0.4 mm^3^, as compared to an average of ∼21 tumors per slide (p<0.01) with average volume of 1.6 mm^3^ in animals not receiving the vaccine (p<0.01), equating to a 75% reduction in both tumor multiplicity and tumor volume (Figures 2G, 2H). It is noteworthy that two out of nine vaccinated animals were completely free of lung tumors (Figure 2G). These results suggest that a KRAS-specific peptides vaccine can inhibit KRAS-driven lung tumorigenesis in prevention setting.

### KRAS vaccination induces T cell-specific immune response and possible intra-antigen epitope spreading

**Figure 3:**
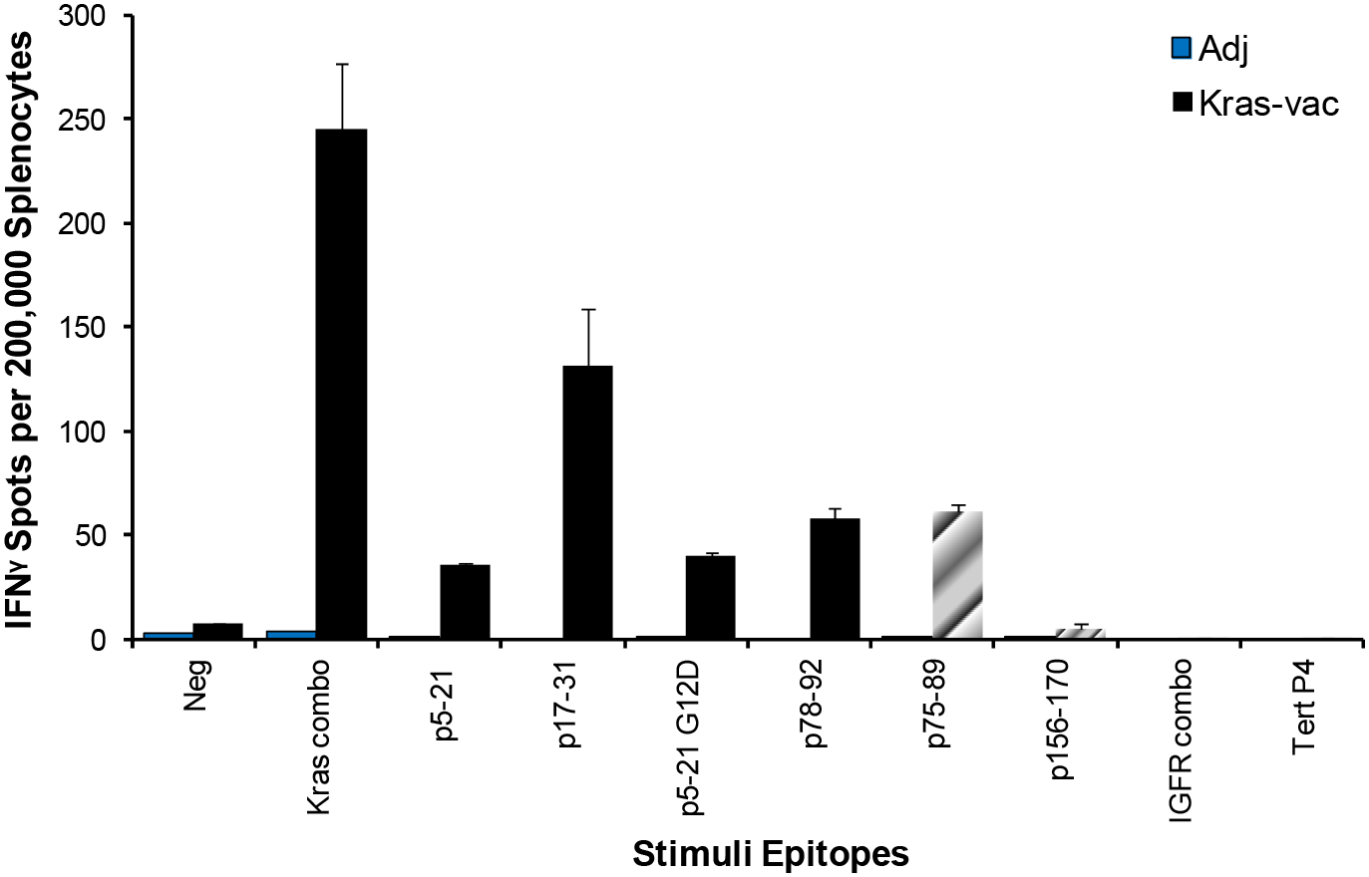
Epitope spreading tested by IFN-γ ELISPOT Mice were vaccinated with the KRAS combination vaccine (p5–21, p5–21 G12D, p17–31, and p78–92). Then splenocytes were collected and pulsed with KRAS combo peptides, each single KRAS peptide, unvaccinated KRAS peptide p75–89, combo IGFR peptides, or Tert peptide. After 72 hours of incubation, the ELISPOT assay was performed, plates were scanned, and spot numbers were statistically analyzed.

In order to examine the specific immunity that confers the protective effect of the KRAS vaccine, an IFN-γ ELISPOT assay of splenocytes isolated from adjuvant alone and vaccinated CCSP-KRAS^G12D^ mice was performed at the end of the study. Results from the ELISPOT assay indicated that vaccinated animals had a robust antigen-specific, IFN-γ-associated immune response to the KRAS vaccine. As shown in Figure 3, splenocytes from the vaccinated mice showed the highest level of IFN-γ response to the mixture of four KRAS peptides used in the vaccine formulation, they also responded to the individual peptides that constituted the vaccine. The mean IFN-γ-secreting cell responses were 216 in the pooled KRAS peptides group, and 36, 40, 116, and 52 to individual peptides p5–21, p5–21 G12D, p17–31, and p78–92, respectively. Interestingly, comparing to A/J mice (Figure 1C), CCSP-KRAS^G12D^ mice (Figure 3) have different response to each single peptide in the ELISPOT assay, which might due to the different MHC-II haplotype between A/J and FVB/NJ strains. Splenocytes from vaccinated animals also developed strong response to non-vaccine KRAS peptide p75–89, which has a 12 amino acid overlap with one peptide p78–92 used in the vaccination (p75–89, Figure 3). This suggests that the current KRAS vaccine may have induced intramolecular epitope spreading. The observed immune response appeared highly specific to the vaccine KRAS peptides, as evidenced by the lack of response in animals that received adjuvant alone; these animals were exposed to the mutated KRAS protein overexpressed in the growing tumor but showed no immunologic reactivity to any peptides tested, including the peptide encompassing the mutant region (p5–21 G12D). There was no evidence of general immune hyperactivity stimulation as none of the animals responded to an irrelevant naïve antigen, further supporting the notion that the immune response detected in the vaccinated group was highly specific to the KRAS vaccine.

### KRAS vaccination increases CD4+/CD8+ cells in lung draining lymph nodes, as well as CD4+ tumor infiltrating lymphocytes

**Figure 4:**
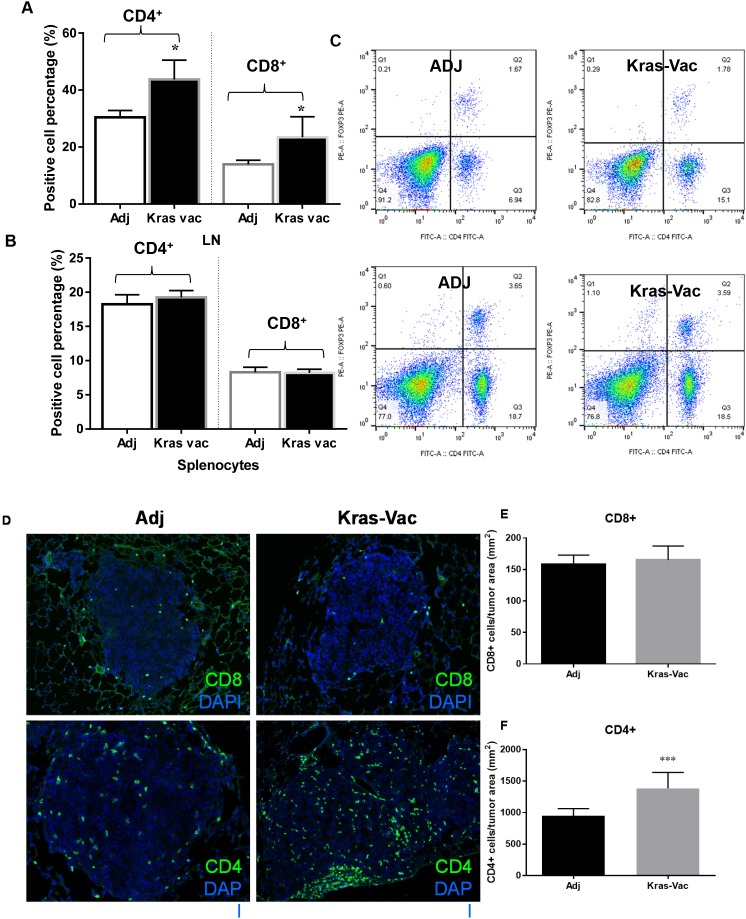
Immunologic Consequences of Vaccination **(A)** KRAS vaccine promotes CD4+ and CD8+ cells in lymph nodes. Representative flow cytometry results for CD4/CD8 cells from mediastinal lung draining lymph nodes (upper panels) and spleen (lower panels). **(B, C)** CD8+ cells expressed as a percentage of total live cells isolated from the lung tumor draining lymph nodes or spleens of adjuvant treated and vaccinated animals at the experimental endpoint (right two columns in B and C); CD4+ cells expressed as a percentage of total live cells isolated from the lung tumor draining lymph nodes or spleens of adjuvant treated and vaccinated animals at the experimental endpoint (left two columns in B and C). **(D)** Tregs were not increased by the KRAS peptide vaccine. Representative flow cytometry data for FoxP3+ cells expressed as a percentage of the total live CD4+ pool isolated from the lungs and spleens of adjuvant treated and vaccinated animals at the experimental endpoint. **(E)** KRAS doesn’t increase the number of CD8+ tumor infiltrating lymphocytes cells in treated animals. **(F)** KRAS increases the number of CD4+ tumor infiltrating lymphocytes in treated animals, *P<0.05, ***P< 0.001.

A detailed flow cytometric analysis of cell surface markers further revealed the differential characteristics of the CD4+ and CD8+ lymphoid populations present both in lymph nodes (Figure 4A) and the spleen (Figure 4B). The overall percentage of CD4+ and CD8+ cells within the lung draining lymph nodes from vaccinated animals trended upward (p=0.16 and 0.056, respectively), while the percentage of Tregs that express CD4 and the FoxP3 transcription factor appeared to remain unaffected (Figure 4C). We further evaluated the number of both CD4+ and CD8+ tumor-infiltrating lymphocytes from representative mice in each group that developed lung cancer (Figures 4D, 4E, 4F). The KRAS vaccine significantly increased the number of intratumoral CD4+ T cells over adjuvant controls, while there was no difference in CD8+ tumor-infiltrating lymphocytes.

### Th1/Th2 cytokine profiles of T cell responses to helper peptide vaccination

**Figure 5:**
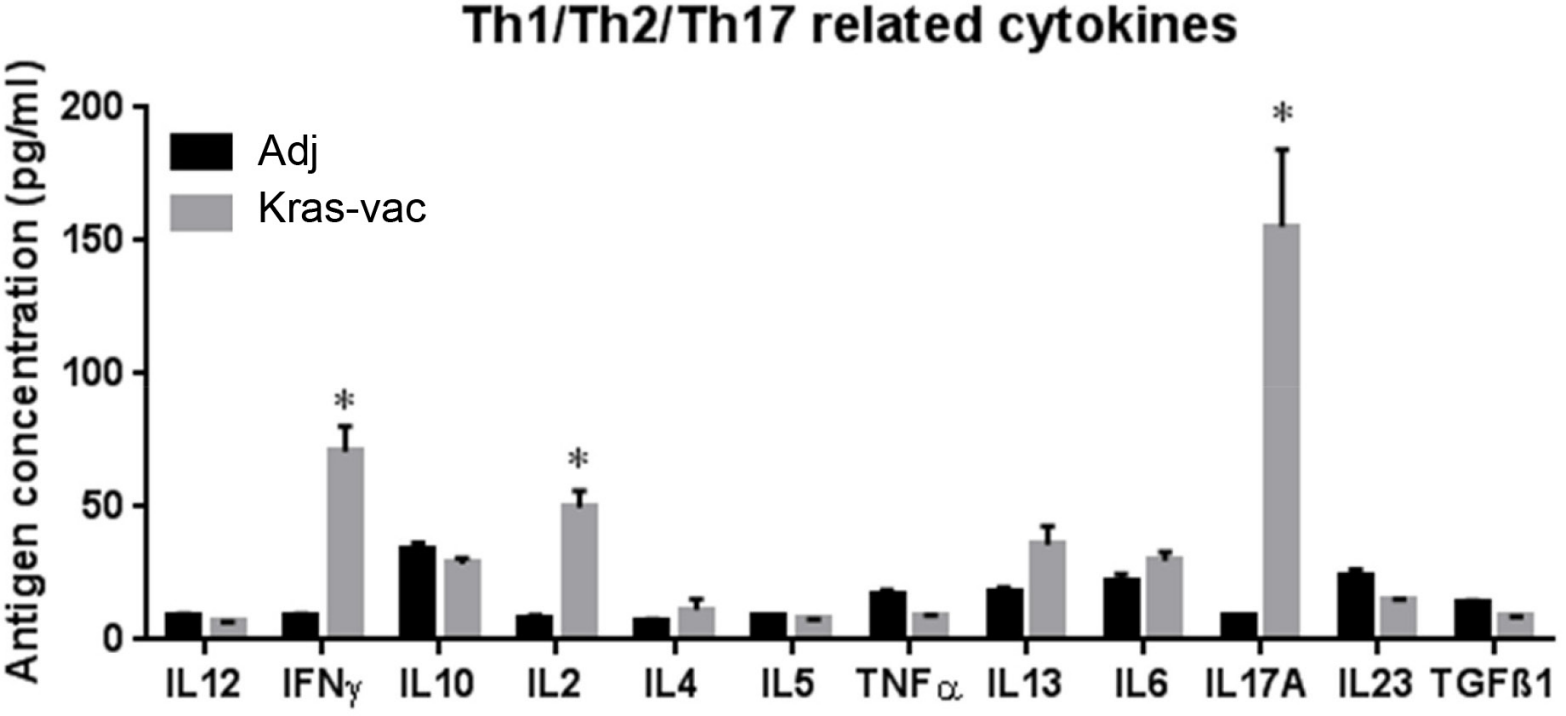
KRAS vaccine induces Th1/Th17 cytokine response Cytokine was analyzed by an ELISArrayKit from QIAGEN. After last boosting vaccination, splenocytes from either KRAS vaccinated or adjuvant alone mice were collected and cocultured with KRAS vaccine for 72 hours; supernatant was collected and assayed with QIAGEN’s ELISArrayKit follow manufacturer’s instruction. Data are shown as the mean ± SE, n=8 (Adj), n=9 (KRAS-Vac), *P<0.05.

Cytokines secreted by splenocytes were measured three days after *in vitro* stimulation with the vaccinating four-peptide pool. Th1 and Th2 cytokine production in response to the KRAS peptide pool is shown in Figure 5. The most abundant individual cytokine detected in response to the KRAS peptide pool was IFN-γ, IL-2, and IL-17A, increasing ∼7-, 5-, and 15-fold from baseline in splenocytes, respectively. Th2 cytokine production, IL-4, Il-5 and IL-13, from the splenocytes in the vaccinated animals as compared to the adjuvant control did not increase notably. These data suggest that the immune responses of Th1, but not Th2, were predominantly elicited by the current KRAS-specific peptide vaccine.

## DISCUSSION

RAS genes (HRAS, NRAS and KRAS) are the most frequently mutated oncogene family in human cancers [23], and is strongly associated with cigarette smoking [24]. For this high-risk population, few strategies are available to mitigate future lung tumor initiation, progression and recurrence. Innovative preventive strategies targeting lung cancer are an urgent need.

In previous preclinical and clinical studies, KRAS mutant-specific CD4 and/or CD8 directed peptide epitopes have been identified [22, 25, 26]. Vaccination with mutant RAS specific peptide or antigen-presenting cells (APCs) loaded with a mutated RAS peptide both generated mutant RAS specific T cell immune response [18, 19, 27–30]. Many of these studies evaluated short peptide-based KRAS vaccines, overwhelmingly in patients with late-stage cancers. Thus, while much can be learned on the safety profiles and immunogenic activity of various KRAS peptides, their antitumor efficacy could not be definitively established.

Constitutively active KRAS expression could also induce immune suppressive environment in developing tumors, which may hamper the assessment of the preventive efficacy of the KRAS vaccine. Here, we started the vaccination before KRAS activation in an inducible CCSP-KRAS murine model, where the vaccination was given before cancer development and thus, theoretically, in the absence of tumor-associated immunesuppression. In contrast to the majority of studies that employed short MHCI restricted peptide vaccines against KRAS, where CD8 cytotoxic T lymohocytes were the major focus, we employed multiple and longer (15–20 amino acid) peptides, which were predicted to have optimal binding affinity to multiple MHC class II alleles. Mutant KRAS has been reported to be presented as both an MHC class I and class II epitope as a foreign antigen; we designed KRAS-derived peptides from hotspots of binding to MHC II in the murine system. Two of the four peptides used in our current KRAS vaccine were derived from outside of the mutation site in codon 12, while the remaining two 17-merpeptides were derived from the region containing either the wild-type or mutated peptide residue at position 12, with a thought to optimize sensitivity to the vaccine.

Several important findings were obtained from the current study. First, the vaccine was strikingly effective in preventing tumor development, causing a roughly 80% decrease in tumor volume in this highly aggressive model. KRAS mutation responsible for various malignancies, such as NSCLC (25%), colorectal cancer (30∼50%), pancreatic cancer (90%), and is strongly associated with cigarette smoking, therefore, our current preclinical data warrant future studies to evaluate the efficacy of the vaccination against many other KRAS-driven cancer types. More importantly, because a substantial level of homology exists between the human and mouse KRAS protein, we were able to identify15–17 amino acid peptides with 100% sequence identity between human and mouse within the hotspot regions, making it possible that these peptides could be translated directly into clinical studies.

Second, we showed that we could get a relatively strong immune response to these 15–20 length peptides using a combination of computer prediction and testing in mice. The observed immunologic response to the KRAS vaccine does not appear to be exclusive to the mutant peptide but rather to the entire wild-type peptides used. Meanwhile, no toxicity was observed in targeting wild-type KRAS. That could be partially due to that KRAS4a is disposable in adult mouse [31], and the endogenous KRAS4b is greatly reduced in these mutant KRAS^G12D^ mice [20]. Therefore, in our current study, the KRAS is an ideal self-protein that is immunogenic and capable of eliciting immunity that will kill KRAS-driven tumors (overexpressing KRAS protein) and not healthy cells (no autoimmunity). Similarly, vaccination with peptide vaccines targeting wild-type EGFR [10] or targeting overexpressed self-antigens (HER2/neu, and IGFBP-2) [9, 21] in mice, or HER2/neu in humans [32] was all well tolerated and showed not toxicity.

Third, the KRAS peptides employed in our study elicited predominantly Th1 T cell responses, without eliciting a strong Th2 response. This suggests that protective immunity against KRAS-driven malignancy may primarily constitute Th1 immune responses. CD4 T cells can differentiate into various Th subpopulations, which can induce and maintain immune responses against tumor antigens, such as Th1, Th2, regulatory T cells (Tregs), and Th17 etc. Th1 subset that produces interferon-gamma (IFN-γ), tumor necrosis factor-alpha (TNF-α) and interleukine-2 (IL-2), regulates cellular immunity and plays a clear antitumor role [33]. In our study, we found the secretion of IFN-γ and IL-2 were significantly increase in KRAS vaccinated mice, indicating KRAS vaccine stimulated CD4 Th1 responses, which is as expected as the peptides were all identified through MHC-II restricted epitope binding algorithms. Our results are also in agreement with previous studies using other MHCII peptide vaccines [34–36]. Furthermore, no increased regulatory T cell population has been found in vaccinated mice may have helped potentiate immune responses to the KRAS peptides. We also noticed in our study a significant increase of IL-17a secretion in KRAS vaccinated mouse splenocytes. IL-17a plays a dual role in the antitumor immunity [37] and is also a key player in the inflammation and tissue destruction associated with autoimmune disease [38]. However, we didn’t observe any toxicity related to this increase, thus, further study will be needed to elucidate the role of IL-17a played in the peptide vaccination.

In summary, we have shown that our KRAS-peptide-based vaccine targeting not only mutant KRAS^G12D^ but other wild-type regions induced robust Th1 immune responses, which were associated with more than 80% inhibition of the KRAS-driven lung tumorigenesis. Meanwhile, no toxicity was observed in targeting wild-type KRAS. Our preclinical data warrant future studies to evaluate the efficacy of the vaccination against early lesions or in the setting of recurrence. Results obtained from such studies will have significant clinical relevance given that KRAS is the gene most frequently mutated across many cancer types.

## MATERIALS AND METHODS

### Mice

Inducible TetOKRAS mice expressing murine KRAS with G12D mutation on FVB background were obtained from the NCI Mouse Models Consortium, and were then crossed with mice expressing the Tet-on Clara Cell Secreted Protein (CCSP) on the A/J background to permit tissue specific inducible expression of the transgene. For all experiments reported herein, only the F1 generation that harbor both KRAS^G12D^ and CCSP were used. All mice were housed in the Biomedical Resource Center at the Medical College of Wisconsin, Milwaukee, WI. All procedures were approved by the institutional animal care and use committee (IACUC).

### Scoring system for the prediction of MHC class II binding epitopes

We and others have shown that peptides that score highly across multiple algorithms are most likely to yield strong immune responses. Therefore, we used the same multi-scoring system as previously described [21]. Briefly, to identify KRAS-specific MHC class II epitopes that have optimal binding affinity and promiscuity across multiple alleles, the following algorithms were used for prediction: SYFPEITHI (Institute for Cell Biology, Heidelberg, Germany), IEDB (www.iedb.org), and Rankpep (Harvard, Boston, MA).

The 11 peptides described in this study were selected as follows. For each available MHC Class II allele from the three algorithms, 20 peptide sequences were initially selected solely on the basis of the rank order of the predicted binding affinity. The sequences are approximately 15 amino acids in length. Individual amino acids for each selected peptide were assigned a score, with 1 being an amino acid contained in a peptide sequence that ranked highest for predictive binding affinity across multiple algorithms. Scoring individual amino acids accounted for the multiple peptides overlaps occurring within and across algorithms. The scores (S) for each amino acid were summed up across the multiple MHC Class II alleles from all three algorithms. Then, the number (N) of MHC class II alleles, for which each amino acid was predicted to have high affinity binding, was counted. The final score for each amino acid was calculated by multiplying S and N. For ease of identifying the most potentially immunogenic segments of the KRAS protein, each amino acid was assigned a color (from dark red to light blue) based on its final score percentile, with dark red being highest at ≥ 75% and light blue the lowest at <10% (Figure 1A). Thus, the dark red color corresponds to a sequence where multiple peptides scored highly within an algorithm as well as across algorithms. Light blue represents sequences that are the least potentially immunogenic of all predicted high binding peptides. KRAS peptides were synthesized by Genemed Synthesis Inc. (South San Francisco, CA), purified by high-performance liquid chromatography, and characterized by mass spectrometry for use in all experiments.

### Vaccine preparation and immunization

Mice were vaccinated with 50 μg of each peptide. Phosphate buffered saline (PBS) was added to bring the total volume to 50 μl/mouse. An equal amount of adjuvant (Complete Freund’s Adjuvant or Incomplete Freund’s Adjuvant) was added to bring the total volume to 100 μl/mouse. Mice were injected subcutaneously on the shoulder at 7 weeks-of-age, and boosting vaccination was given every two weeks for the first three boosting, and every four weeks for the last three boosting as shown in Figure 2A. Transgene KRAS was initiated with Dox diet (625 mg/kg diet) one week after the fourth vaccination, and Dox diet was given throughout of the study.

### ELISPOT assay

Cell suspensions from whole spleens were filtered through a 70 μm cell strainer (BD) and subjected to red blood cell lysis using ACK lysis buffer. 1.5∼3.0 × 10^4^ cells were plated into individual wells of a MAIPS4510 Multiscreen 96-well plate previously coated with anti-interferon γ detection antibody and containing media with either peptide, positive control (concanavalin A) or negative control (HIV peptide, or no antigen). After 72 hours, plates were washed and a secondary antibody (BD) was added and incubated on the plate overnight at 4°C. Wells were then washed with PBS and HRP streptavidin was added. Following 1hour incubation, the plate was developed using AEC substrate for between 5 to 25 minutes. The plate was subsequently gently washed under cold running tap water. When dry, an automated plate reader system (CTL Technologies) was used to image the plates and quantify spot number.

### Magnetic resonance imaging

Mice were imaged using a 9.4T MRI (Bruker, Billerica, MA) with a custom birdcage style quadrature coil (Doty Scientific, Columbia, SC). Mice were anesthetized with isoflurane for the duration of the imaging procedure. Mice were induced at 2.5% isoflurane and maintained at 1.0-1.5%. The heart rate, body temperature and respiratory rate were continuously monitored throughout imaging. Both respiratory and cardiac gating using an electrocardiogram were used to ensure that images were consistently acquired during latent periods of the respiratory cycle and at a consistent point during the cardiac cycle. Tumors were imaged using a multi-slice, multi-echo acquisition (MSME). Images were acquired using the following parameters; TE=8.07 ms, TR≥400 ms (variable), matrix=128 X 128, 1 average, 20 axial slices.

### Lung tumor counting using H&E staining

Mouse lung samples from CCSP-KRAS^G12D^ mice were inflated and formalin fixed and processed (Sakura Tissue Tek VIP5) for paraffin embedding. After paraffin embedding, samples were sectioned at 4 μm (Microm HMS355S) onto poly-l-lysine coated slides and air dried at 45°C overnight prior to immunohistochemistry or H&E staining.

H&E stained slides were scanned using the NanoZoomer slide scanner (Hamamatsu). Subsequently, NanoZoomer software was used and tumor regions were specifically highlighted and measured. Three slides were selected per mouse for analysis, corresponding to a ventral, midline and dorsal region of the lung.

### Flow cytometry

Mesenchymal lymph nodes, mouse lung or spleen were minced and processed to single cell suspensions. Single cells were evaluated using flow cytometry for expression of surface markers CD4, CD8, CD44, CD62L and CD25 (eBioscience), as well as intracellular staining for FoxP3 (eBioscience). Stained cells were fixed in 1% paraformaldehyde and were permeabilized following the manufacturer’s instructions to evaluate the expression of intracellular targets (FoxP3). Flow cytometry was conducted using an LSR-II flow cytometer (BD). Data was analyzed using FlowJo software (Tree Star).

### Cytokine analysis

Mouse Th1/Th2/Th17 Cytokines Multi-AnalyteELISArray™ Kits (Qiagen) were used for cytokine analysis; it analyzes a panel of 12 cytokines involved in T helper cell biology. The cytokines represented by this array are IL2, IL4, IL5, IL6, IL10, IL12, IL13, IL17A, IL23, IFNγ, TNFα, and TGFβ1. Splenocytes from different groups of mice were stimulated with different peptides for 72 hours, and then supernatant was collected and assayed based on the manufacturer’s instructions.

### Evaluation of tumor-infiltrating T cells

Tumors were frozen in Tissue-Tek OCT and stored at -80°C. Frozen tumors were then sectioned (8 μm), fixed in 75%/25% acetone/methanol for 5 minutes, and washed using PBS. Slides were incubated with normal goat serum (10% in PBS) for 1 hour at room temperature, washed, and incubated with rat anti-mouse CD8 (AbDSerotec) at 1:100 dilution in 10% goat serum/PBS overnight at 4°C. After washing, Alexa Fluor 488 goat anti-rat secondary antibody (Invitrogen) was added to the slides (1:1000) for one hour at room temperature. Prolong Gold antifade with 4´, 6-diamidino-2-phenylindole (DAPI) mounting media (Invitrogen) was added after an additional wash and cover slips were attached. Positive cells and DAPI stained nuclei were counted in three random high-powered microscopic fields per slide and expressed as a mean. The number of positive cells in the field was expressed as # of CD8^+^ cells per mm^2^ tumor area. Data are shown as the mean and SEM for 3 mice/group.

### Statistical analysis

All *in vitro* assays were performed at least in triplicate. Five to nine mice per group were used for the *in vivo* studies. A two-tailed Student’s t-test was used to evaluate differences between the control and each treatment group. *P< 0.05, **P< 0.01 and ***P< 0.001 were considered statistically significant.

## SUPPLEMENTARY MATERIALS FIGURE AND TABLE


