# Supplementary material for: Immunoprevention of KRAS-driven lung adenocarcinoma by a multipeptide vaccine
Source: Oncotarget. 2017 Aug 1;8(47):82689–99. doi: 10.18632/oncotarget.19831 (PMC5669920; doi:10.18632/oncotarget.19831)
Supplement: Supplementary file 1 [file oncotarget-08-82689-s001.pdf]

Immunoprevention of KRAS-driven lung adenocarcinoma by a multipeptide vaccine

SUPPLEMENTARY MATERIALS

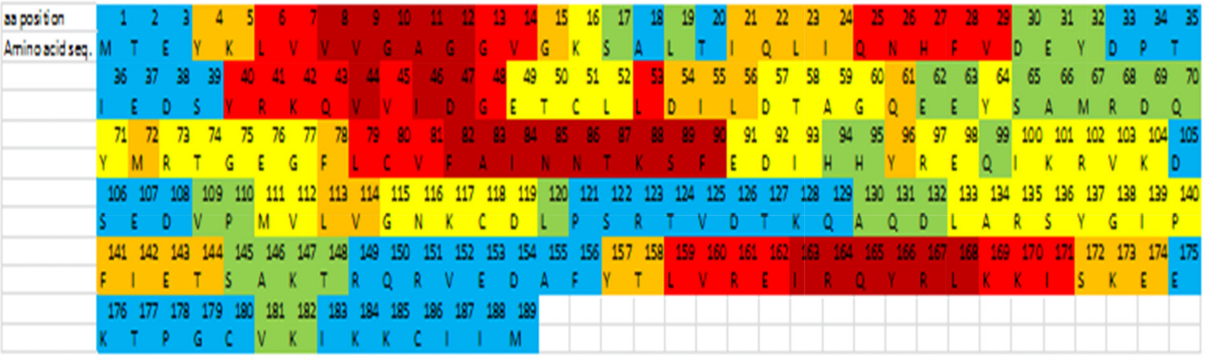

Supplementary Figure 1: Immunogenic heatmap for human Kras with common 14 MHC clss II alleles.

Supplementary Table 1: Homology and sequence of peptides derived from Ki-Ras protein

	Kras peptides	Peptide sequence	% Homology with human Kras
G12D	**p5-21 G12D**	**KLVVVGADGVGKSALTI**	**100**
61	**p5-21**	**KLVVVGAGGVGKSALTI**	**100**
62	**p5-19**	**KLVVVGAGGVGKSAL**	**100**
63	**p17-31**	**SALTIQLIQNHFVDE**	**100**
64	**p11-25**	**AGGVGKSALTIQLIQ**	**100**
65	**p33-47**	**DPTIEDSYRKQVVID**	**100**
66	**p40-54**	**YRKQVVIDGETCLLD**	**100**
67	**p75-89**	**GEGFLCVFAINNTKS**	**100**
68	**p78-92**	**FLCVFAINNTKSFED**	**100**
69	**p130-144**	**AQELARSYGIPFIET**	**93.9**
70	**p135-149**	**RSYGIPFIETSAKTR**	**100**
71	**p154-168**	**DAFYTLVREIRKHKE**	**73.3**
72	**p156-170**	**FYTLVREIRKHKEKM**	**66.7**
